# Preliminary assessment of free radical scavenging, thrombolytic and membrane stabilizing capabilities of organic fractions of *Callistemon citrinus* (Curtis.) skeels leaves

**DOI:** 10.1186/s12906-016-1239-1

**Published:** 2016-07-26

**Authors:** Farhana Ahmed, Mohammad Sharifur Rahman

**Affiliations:** Phytochemical Research Laboratory, Department of Pharmaceutical Chemistry, Faculty of Pharmacy, University of Dhaka, Dhaka, 1000 Bangladesh

**Keywords:** *Callistemon citrinus*, Myrtaceae, Free radical scavenging, Membrane stabilization, Thrombolytic, Antiinflammatory, Antioxidant, Herbal

## Abstract

**Background:**

*Callistemon citrinus* (Curtis.) (Family- Myrtaceae) is a popular evergreen shrub in Bangladesh. In the present study, the leaves of this plant have been assessed comprehensively for free radical scavenging, thrombolytic and membrane stabilizing activities.

**Methods:**

The leaves were collected, powdered and extracted with methanol. The extract was then concentrated and successively fractionated into petroleum ether, carbon tetrachloride, chloroform and aqueous soluble fractions. The extractives were investigated for free radical scavenging, thrombolytic and membrane stabilizing activities.

**Results:**

In case of 1,1 diphenyl-2-picrylhydrazyl (DPPH) and hydrogen peroxide radical scavenging assays, the crude methanol extract of the leaves showed the highest free radical scavenging activity among the tested materials including standard ascorbic acid (*p* = 0.0000). Besides, this extract was also found significantly rich (*p* = 0.0000) in phenolics and flavonoids compared to other organic fractions. In thrombolytic study, the petroleum ether fraction exhibited significantly stronger thrombolysis (*p* = 0.024) than other leaf extractives but was weaker than the standard streptokinase. In membrane stabilizing assay, the activity of chloroform fraction was similar to that of standard acetylsalicylic acid (*p* = 1.000) in hypotonic solution induced hemolysis. However, membrane stabilization activity of this chloroform fraction was found significantly stronger than that of the standard (*p* = 0.0000) in heat induced hemolysis.

**Conclusion:**

This study has revealed the medicinal capabilities of different organic fractions of *C. citrinus* displaying free radical scavenging, thrombolysis and membrane stabilizing antiinflammatory potentials. Further bioactivity guided isolation is required to obtain pharmacologically secondary metabolites.

## Background

Plants are the important sources of various pharmacologically active compounds [1]. Among World Health Organization (WHO) enlisted 252 basic and essential drugs, 11 % are solely of plant origin [2]. It has been assessed that there are more than 250,000 flower plant species in the world [3]. Exploration of bioactivities of medicinal plants aids to develop phytotherapeutic agents [4]. WHO has exclusively acknowledged the significance of herbal medicine for health care system and published many strategies, guidelines and standards for these botanical medicines [5].

Free radicals produced in living cells might result in oxidative stress, which might trigger a number of diseases. Antioxidant emerges as savior from this condition [6]. Besides, some synthetic antioxidants such as butylated hydroxytoluene and butylated hydroxyanisole have been reported to be unsafe for human health. Thus, the pursuit for effective, nontoxic and natural antioxidants has been intensified in recent years [7]. Mortality rate from thrombotic diseases such as myocardial or cerebral infarction is increasing recently. This situation might become more challenging in the future due to obesity, diabetes, metabolic syndromes, etc. [8]. Currently, much effort has been concentrated on the discovery of natural products as effective antithrombotic drugs [9]. Inflammatory processes are involved with many severe conditions, such as neurodegenerative diseases, asthma, chronic inflammatory bowel diseases, rheumatoid arthritis, type 2 diabetes, cancer, etc. [10]. The currently available antiinflammatory drugs include nonsteroidal antiinflammatory drugs, glucocorticoids, immunosuppressant drugs and biologicals. Despite these, therapy is often hindered by side effects. Thus, the discovery of new antiinflammatory drugs is still a need of the present time [11].

*C. citrinus* (Curtis.) belongs to the family Myrtaceae. It is an evergreen shrub known as Red bottle-brush or Lemon bottle-brush and available all over Bangladesh. The flower spikes of bottle-brushes form in spring and summer and are made up of a number of individual flowers. This is a woody plant and can be 3 m to 4.6 m long [12]. This herb is popular as folk medicine to treat diarrhea, dysentery, rheumatism, cough, bronchitis, etc. [13, 14]. It has already been reported that this plant has antimicrobial [15], relaxant [16] and cardioprotective [17] properties. Previous phytochemical investigations led to the isolation of various terpenoids, flavonoids, etc. A herbicidal compound, nitisinone, was also isolated from this plant [18].

In the present study, the leaves of *C. citrinus* were assessed comprehensively for its free radical scavenging, thrombolytic and membrane stabilizing activities.

## Methods

### Collection and identification of plant

The leaves of *C. citrinus* were collected from Dhaka on August, 2013. The plant was formally identified by an expert taxonomist at the Bangladesh national herbarium (BNH), Mirpur Road-1, Dhaka (http://www.bnh.gov.bd). BNH is a government institute, which provides plant identification service nationally. For the current experimental plant, an accession number (DACB −38386) was provided by BNH after identification. The voucher specimen of the plant has been preserved there for future reference.

### Extraction and fractionation

**Fig. 1:**
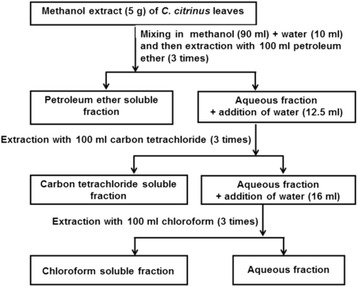
Fractionation. Schematic representation of the modified Kupchan partitioning of methanolic crude extract of the leaves of *C. citrinus*

The collected leaves were dried and powdered. 1 kg of the powdered materials was soaked separately in 5 L of methanol at room temperature for 7 days. The extract was filtered through cotton plug and concentrated with a rotary evaporator. An aliquot (5 g) of the concentrated methanol extract was fractionated by modified Kupchan method [19] to provide petroleum ether, carbon tetrachloride, chloroform and aqueous soluble fractions in a successive manner (Fig. 1). Subsequent evaporation of solvents yielded petroleum ether (3.22 g), carbon tetrachloride (1.12 g), chloroform (0.10 g) and aqueous (0.25 g) soluble materials, respectively.

### Preliminary phytochemical screening

The presence of steroids, quinones, flavonoids and alkaloids was examined by the reported protocol of Joshi et al., 2013 and Kchaou et al., 2016 [20, 21].

- *Test for triterpenoids and steroids*. 2 mg of extract was mixed well with 1 ml of acetic anhydride (Merck, Germany) followed by boiling and cooling. Then, 1 ml of concentrated sulphuric acid (Merck, Germany) was added. A brown ring was formed at the junction of two layers. Formation of green and red color indicated the presence of steroids and triterpenoids, respectively.
- *Test for quinones*. 2 mg of extract and 2 ml of sodium hydroxide (0.1 M) (Merck, Germany) were mixed well. Pink or violet or red color formation indicated the presence of quinones.
- *Test for alkaloids*. 2 mg of extract was mixed properly with 0.5 ml of 1 % hydrochloric acid (Merck, Germany) followed by a few drops of Mayer’s reagent, which was composed of a aqueous solution of 1.36 (w/v) mercuric chloride (Sigma-Aldrich, USA) and 5 % (w/v) potassium iodide (Sigma-Aldrich, USA). The formation of cream or yellow precipitate indicated the presence of alkaloids.

### DPPH free radical scavenging activity

The free radical scavenging activity of the extract was determined based on the scavenging activity of the stable 1,1 diphenyl-2-picrylhydrazyl (DPPH) free radical [22]. In brief, methanol solutions of the test materials (2 ml) were prepared at different concentrations and then mixed well with 3 ml of a DPPH (Sigma-Aldrich, USA) methanol solution (20 μg/ml). Reaction mixtures were kept in dark for 20 min at room temperature. Then, absorbance was determined at 517 nm (UV-1800 UV–VIS Spectrophotometer, Shimadzu, Japan). Here, ascorbic acid was used as standard. The percentage inhibitory activity (I%) was calculated from,

$$ I\%=\left(1{\textstyle \hbox{-} }{A}_{sample}/{A}_{blank}\right)\times 100 $$

Where, A_blank_ is the absorbance of the control reaction. Extract concentration providing 50 % inhibition (IC_50_) was calculated from the graph plotted inhibition percentage against extract concentration.

### Hydrogen peroxide free radical scavenging activity

The free radical scavenging activity was determined by the scavenging activity of the hydrogen peroxide free radical [23]. In this study, hydrogen peroxide (Merck, Germany) of 43 mM was prepared in phosphate buffer saline (pH 7.4). Ascorbic acid (standard) (Sigma-Aldrich, USA) and the extract solution were prepared separately at concentration from 500 to 15.625 μg/ml. Aliquot of either standard or extract solution (3.4 ml) was mixed with 0.6 ml of hydrogen peroxide solution. The reaction mixtures were incubated at room temperature for 10 min and the absorbance was determined at 230 nm (UV-1800 UV–VIS Spectrophotometer, Shimadzu, Japan). The percentage inhibitory activity (I%) was calculated from,

$$ I\%=\left(1{\textstyle \hbox{-} }{A}_{sample}/{A}_{blank}\right)\times 100 $$

Where, A_blank_ is the absorbance of the control. IC_50_was calculated from the graph plotted inhibition percentage against extract concentration.

### Total phenolic content determination

Total phenolic content of the extractives was measured by using Folin-Ciocalteu reagent (Merck, Germany) as an oxidizing agent and gallic acid (Merck, Germany) as a standard [24]. In this assay, 0.5 ml of the extract (2 mg/ml) in water was mixed with 2.5 ml of Folin-Ciocalteu reagent (10 times diluted with water) and 2 ml of 7.5 % w/v sodium carbonate (Merck, Germany) solution and incubated for 20 min in room temperature. The absorbance was measured at 760 nm using a UV-visible spectrophotometer (UV-1800 UV–VIS Spectrophotometer, Shimadzu, Japan). Total phenolic content was quantified by calibration curve obtained from measuring the known concentrations of gallic acid (0–100 μg/ml). It was expressed as mg of GAE (gallic acid equivalent)/g of the dried extract.

### Total flavonoid content determination

Total flavonoid content was measured by aluminum chloride colorimetric assay described earlier by Kchaou et al., 2016 [21]. 1 ml of 2 % aluminum chloride (Sigma-Aldrich, USA) solution was added to an aliquot (2 mg/ml methanol) of extract. The solution was mixed well and incubated for 15 min. The absorbance was measured by UV-visible spectrophotometer (UV-1800 UV–VIS Spectrophotometer, Shimadzu, Japan) against prepared reagent blank at 430 nm. Total flavonoid content was quantified by calibration curve obtained from measuring the known concentrations (0–100 μg/ml of methanol) of quercetin (Sigma-Aldrich, USA). It was expressed as mg of QE (quercetin equivalents)/g of the dried extract.

### Thrombolytic activity

For in vitro thrombolytic assay [25], 5 ml venous blood were drawn from healthy volunteers and then dispensed in pre-weighed sterile microcentrifuge tubes (0.5 ml/tube). Incubation of them at 37 °C for 45 min allowed forming clot. The developed serum was removed without disturbing the clot and each tube was again weighed to measure the clot weight. Leaf extractives (2 mg/100 μl water) were added in the tubes. 100 μl (equivalent to 30,000 I.U.) of streptokinase (Altepase®, Beacon pharmaceuticals Limited, Bangladesh) and 100 μl of distilled water were used as positive and negative control, respectively. After 90 min incubation of the tubes at 37 °C, the developed fluid from the clot was discarded very carefully and tubes were weighed again. Percentage of lysis of clot was expressed as:

$$ \%thrombolysis=\left( weightofreleasedclotaftertreatment/ weightofclotbeforetreatment\right)\times 100 $$

### Membrane stabilizing activity

Hypotonic solution- and heat- induced hemolysis methods were used for conducting membrane stabilizing assay [24, 26]. For erythrocyte suspension preparation, 5 ml of whole blood was collected from healthy human volunteers in a tube containing dipotassium salt of EDTA (Merck, Germany) at a concentration 2.2 mg/ml of blood. It was centrifuged and blood cells were successively washed three times with equal volume of supernatant by isotonic solution (154 mM NaCl) in 10 mM sodium phosphate (Merck, Germany) buffer (pH 7.4) through centrifugation (10 min at 3000 g). The cells were resuspended in the same volume of isotonic buffer solution.

- *Hypotonic solution*-*induced hemolysis*. 500 μl of erythrocyte suspension was mixed together with 5 ml of hypotonic solution (50 mM NaCl) in 10 mM sodium phosphate buffered saline (pH 7.4) containing the leaf extractive (2 mg/ml) or reference drug, acetylsalicylic acid (0.1 mg/ml) (Sigma-Aldrich, USA). The control sample was consisted of 0.5 ml of erythrocyte mixed with hypotonic-buffered saline alone. The mixture was incubated for 10 min at room temperature and centrifuged for 10 min at 3000 g. Later, the optical density (OD) of the supernatant was measured at 540 nm (UV-1800 UV–VIS Spectrophotometer, Shimadzu, Japan) for calculating the percentage inhibition of hemolysis using the following equation-
  $$ \%Inhibitionofhemolysis=\left\{\left(O{D}_{control}-O{D}_{testsamples}\right)/O{D}_{control}\right\}\times 100 $$
- *Heat*-*induced hemolysis*. 5 ml of isotonic buffer having aliquot of leaf extractive (2 mg/ml) or reference drug, acetylsalicylic acid (0.1 mg/ml) (Sigma-Aldrich, USA) was placed into centrifuge tube. The erythrocyte suspension (30 μl) was then added to the tube and mixed gently. One set of the tubes along with control samples were placed in water bath for incubation at 54 °C for 20 min, while the other set of the tubes were kept at 0–5 °C in an ice bath. Later, the reaction mixture was centrifuged for 3 min at 1300 g and the absorbance of the supernatant was measured at 540 nm (UV-1800 UV–VIS Spectrophotometer, Shimadzu, Japan). The percentage inhibition of hemolysis was calculated according to the equation:

$$ \%Inhibitionofhemolysis=\left\{1{\textstyle \hbox{-}}\left(O{D}_{heatedtestsample}{\textstyle \hbox{-} }O{D}_{unheatedtestsample}\right)/\left(O{D}_{heatedcontrolsample}{\textstyle \hbox{-} }O{D}_{heatedtestsample}\right)\right\}\times 100 $$

### Statistical analysis

Three replicates (*n* = 3) of each sample were used for statistical analysis and the values are reported as mean ± standard deviation (SD). Data were analyzed by statistical package for social science (SPSS) software (Version 20, IBM Corporation, USA) using one-way ANOVA followed by Tukey’s post hoc test for multiple comparisons. The values were considered significantly different at *p* < 0.05.

## Results

### Preliminary phytochemical screening

The result of the preliminary phytochemical screening is shown in Table 1. Steroids and triterpenoids were abundant in petroleum ether and carbon tetrachloride fractions. However, quinones were present moderately in carbon tetrachloride and chloroform fractions. Besides, alkaloids were present in chloroform fraction to a larger extent.

**Table 1:** Preliminary phytochemical screening of different extractives of *C. citrinus* leaves

Test	Crude methanol	Petroleum ether	Carbon tetrachloride	Chloroform	Aqueous
Triterpenes	++	+++	+++	+	-
Steroids	++	+++	+++	-	-
Quinones	++	-	++	++	+
Alkaloids	++	-	++	+++	+

+++ indicates abundant, ++ indicates moderately present, + indicates trace; − indicates absent

### DPPH free radical scavenging activity

**Fig. 2:**
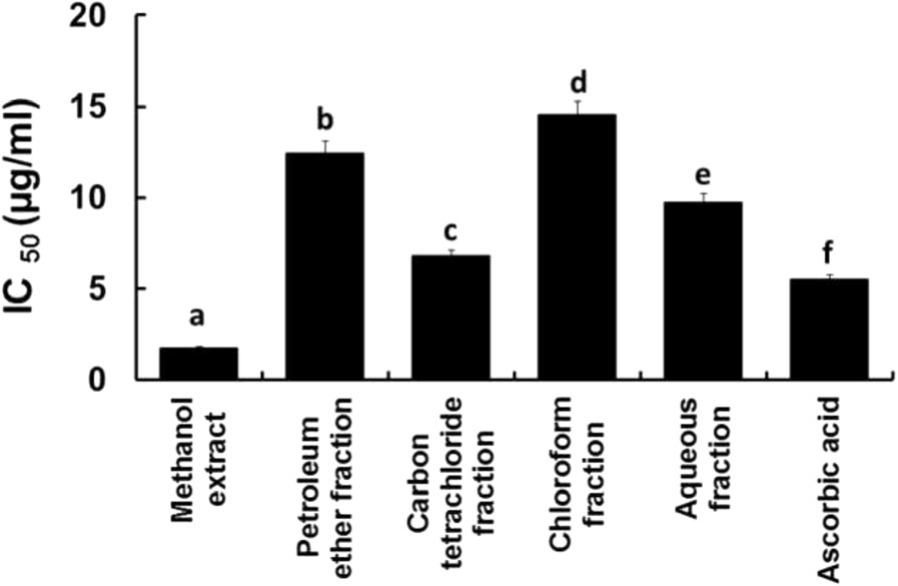
IC_50_ values of the standard and extractives of the leaves of *C. citrinus* in terms of DPPH free radical scavenging capacity. Bars with different letters are significantly different (*p* < 0.05)

The DPPH free radical scavenging activities are illustrated in Fig. 2. Here, the DPPH free radical scavenging activity of the crude methanol extract (IC_50_ value 1.74 μg/ml) was significantly stronger (*p* = 0.0000) than that of the standard drug, ascorbic acid (IC_50_ value 5.47 μg/ml).

The order of activity of the extractives might be mentioned as:

$$ Crude\  methanol > carbon\  tetrachloride > aqueous > petroleum\  ether > chloroform $$

### Hydrogen peroxide free radical scavenging activity

**Fig. 3:**
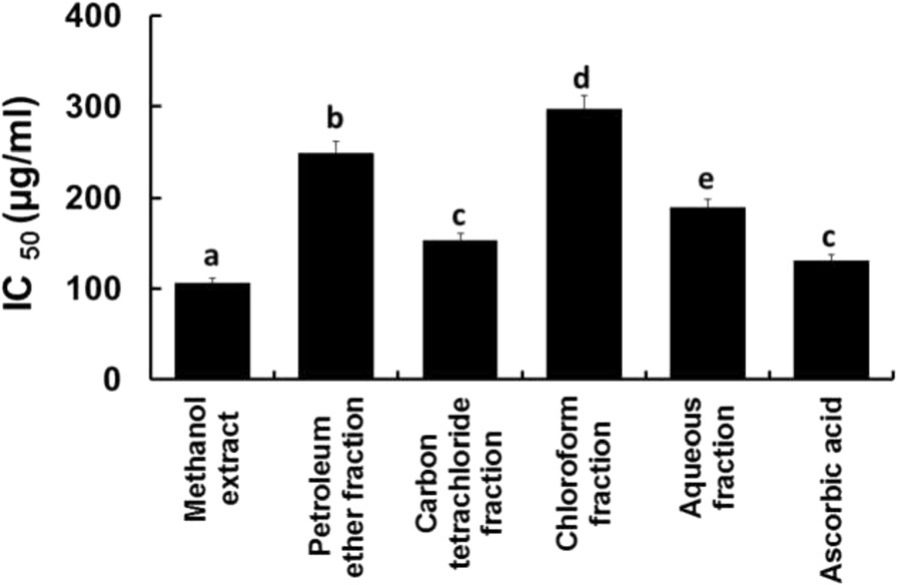
IC_50_ values of the standard and extractives of the leaves of *C. citrinus* in terms of hydrogen peroxide scavenging activity. Bars with different letters are significantly different (*p* < 0.05)

The hydrogen peroxide free radical scavenging activities are shown in Fig. 3. Here, the hydrogen peroxide free radical scavenging activity of the crude methanol extract (IC_50_ value 106.03 μg/ml) was significantly stronger (*p* = 0.047) than that of the standard drug, ascorbic acid (IC_50_ value 131.04 μg/ml). No significant difference (*p* = 0.082) of free radical scavenging activity was seen between the carbon tetrachloride fraction and ascorbic acid.

The order of activity of the extractives might be mentioned as:

$$ Crude\  methanol > carbon\  tetrachloride > aqueous > petroleum\  ether > chloroform $$

### Total phenolic content determination

**Fig. 4:**
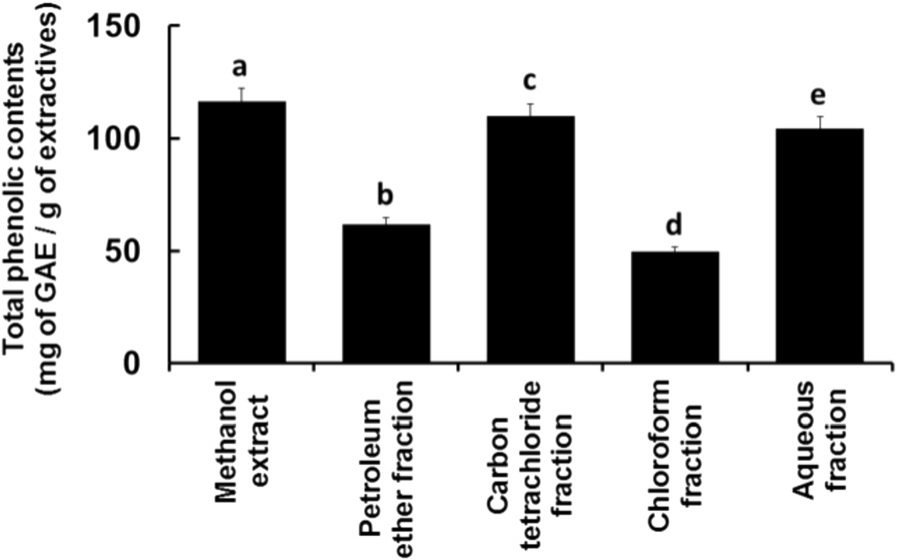
Total phenolic contents of the leaves extractives of *C. citrinus*. Bars with different letters are significantly different (*p* < 0.05)

The phenolic contents of the extractives are shown in Fig. 4. Among all the extractives, a significantly higher level (*p* = 0.0000) of phenolic content was determined in the crude methanol extract (116.36 mg of GAE/g of extractive).

### Total flavonoid content determination

**Fig. 5:**
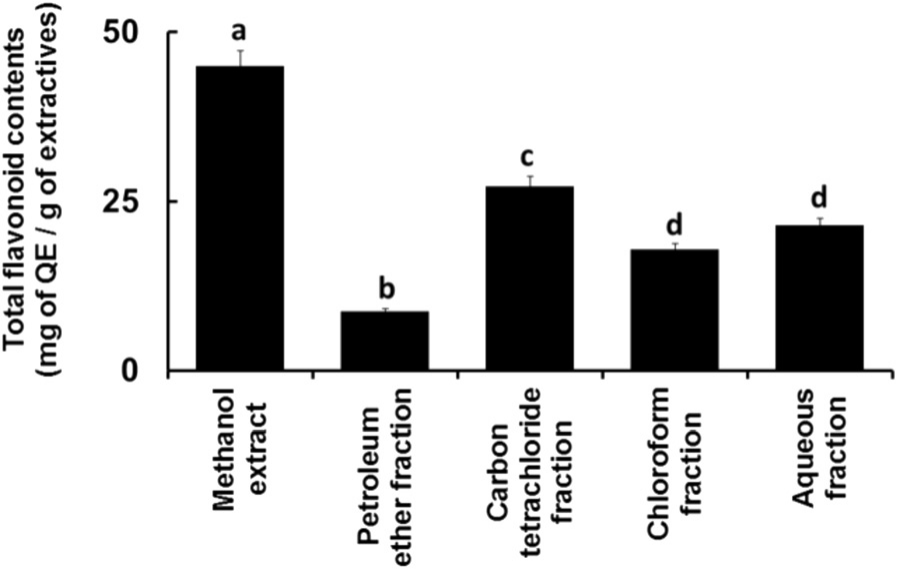
Total flavonoid contents of the leaves extractives of *C. citrinus*. Bars with different letters are significantly different (*p* < 0.05)

The flavonoid contents of the extractives are shown in Fig. 5. The level of flavonoids in crude methanol extract (45.03 mg of QE/g of extractive) was significantly higher (*p* = 0.0000) than other tested extractives.

### Thrombolytic activity

**Fig. 6:**
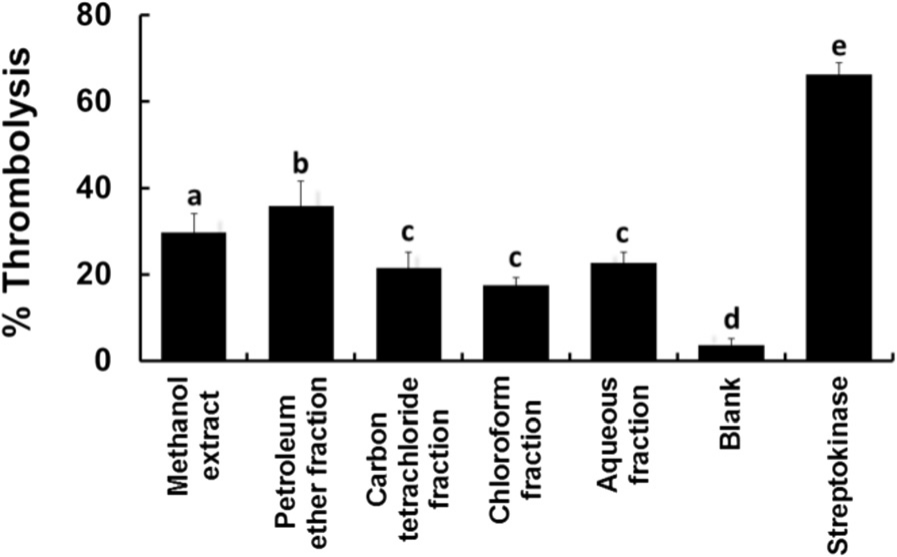
Effect of different extractives of leaves of *C. citrinus* on thrombolysis. Bars with different letters are significantly different (*p* < 0.05)

The thrombolytic activities of the leaf extractives are shown in Fig. 6. The highest activity was displayed by the petroleum ether fraction (35.95 %), which was significantly stronger (*p* = 0.024) than other tested extractives. However, thrombolytic activity of this fraction was weaker than that of the standard streptokinase.

### Membrane stabilizing activity

**Fig. 7:**
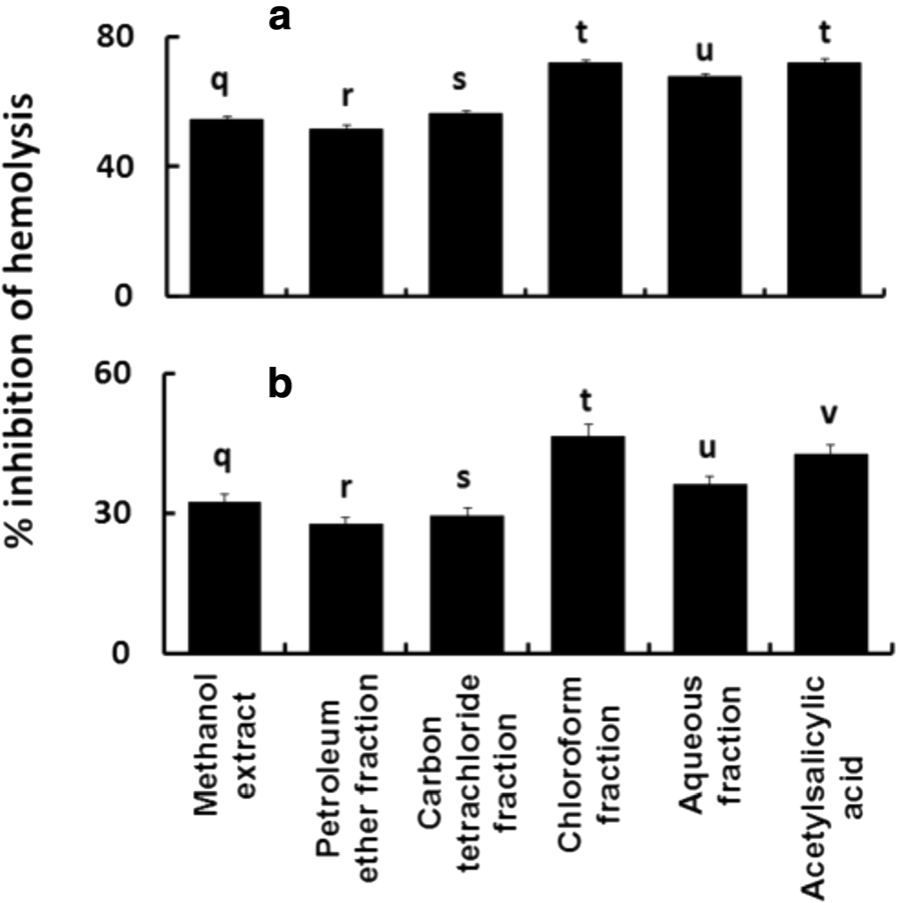
Effect of different extractives of *C. citrinus* on hypotonic solution induced (a) and heat induced (b) hemolysis of erythrocyte membrane. Bars with different letters are significantly different (*p* < 0.05)

The membrane stabilizing activities are displayed in Fig. 7. In case of hypotonic solution-induced hemolysis (Fig. 7a), the maximum level of membrane stabilizing activity (71.84 %) was observed for the chloroform fraction, which was not significantly different (*p* = 1.000) from the action of the standard drug, acetylsalicylic acid (71.98 %). In case of heat-induced hemolysis (Fig. 7b), the chloroform fraction exhibited highest protection (46.65 %) to the membrane, which was significantly stronger (*p* = 0.0000) than that of the standard drug, acetylsalicylic acid (42.61 %).

## Discussion

Traditional medicine has gained popularity in all regions of the developing world for meeting some primary health care needs and is also rapidly spreading in industrialized countries [27]. WHO has estimated that 25 % of modern medicines are made from plants first used traditionally [28]. It has been assessed that the global market for herbal medicines is currently over 60 billion United States dollar annually and growing steadily [29]. Even in the present era of synthetic medicine, drug discovery research based on herbal medicines is successful to a significant extent [3, 30]. With this view, *C. citrinus*, a plant of Bangladesh, has been studied here utilizing crude methanol extract of leaves and its organic fractions.

Free radicals are produced in human body by various endogenous systems, exposure to different physiochemical conditions or pathological states [7]. In excess, they can damage cellular macromolecules including carbohydrates, proteins, lipids and nucleic acids. Ultimately, they promote inflammatory damages, cataract, cancer, aging, thrombus formation and many other associated problems [31]. To counteract these disorders, intake of antioxidants are very essential [6]. In DPPH and hydrogen peroxide free radical scavenging assays, the methanol extract scavenged free radicals prominently among all the tested materials including the standard drug, ascorbic acid. The carbon tetrachloride fraction also had a tendency to inhibit free radical production to an extent in both assays (Figs. 2 and 3).

The analysis of crude methanol extract using UV-visible spectroscopic technique in the present study demonstrated the abundance of phenolics and flavonoids than other extractives (Figs. 4 and 5). Besides, its carbon tetrachloride fraction also displayed noticeable level of phenolic and flavonoid contents. These findings might partially explain the reason for stronger free radical scavenging ability of the crude methanol extract and its carbon tetrachloride fraction [32, 33].

However, thrombosis occurs due to the imbalance between thrombogenic factors and protective mechanisms, which leads to many vascular complexities including stroke, myocardial infarction, deep vein thrombosis, portal vein thrombosis, renal vein thrombosis, etc. [34]. Tissue plasminogen activator, urokinase, streptokinase, etc. are used abundantly for treating thrombotic disorders but they are not beyond all limitations. Better thrombolytic agent is a necessity of time [35, 36]. In this study, the petroleum ether soluble fraction displayed the highest thrombolytic activity among the tested extractives of the leaves (Fig. 6). It has been reported earlier that terpenoids might have significant potentials of displaying thrombolytic activity [37, 38]. Leaves of *C. citrinus* were reported previously to have some terpenoids [18]. Besides, a preliminary phytochemical screening of this study (Table 1) also indicated the presence of abundant terpenoids in petroleum ether fraction. This might be one of the reasons for exhibiting noticeable thrombolytic activity of the petroleum ether fraction among the tested extractives.

On the other hand, the membrane stabilization assay using erythrocytes is used as a common tool to screen antiinflammatory materials. The erythrocyte membrane is comparable to the lysosomal membrane and its stabilization acts as a parameter to indicate the ability of an extract to stabilize the lysosomal membrane as well [26]. Maintaining stability of the lysosomal membrane is vital to limit the inflammatory response by inhibiting the release of lysosomal constituents of activated neutrophil such as bactericidal enzymes and proteases, which cause additional tissue inflammation and damage upon extracellular release [39]. Exposure of erythrocytes to hypotonic medium, heat, methyl salicylate, phenylhydrazine, etc., results in membrane lysis [40]. In the present study, the chloroform soluble fraction showed the highest membrane stabilizing activity in both heat- and hypotonic solution induced hemolysis among the plant extractives (Fig. 7). It was equivalent to the membrane stabilizing action of the standard drug, acetylsalicylic acid in the hypotonic solution induced hemolysis assay and more effective than the standard drug in heat induced hemolysis assay. Numerous flavonoids and alkaloids have been reported previously to display antiinflammatory activity [41, 42]. Chloroform fraction of the leaves of *C. citrinus* was also found to contain flavonoids (Fig. 5) and alkaloids (Table 1). The persuasive membrane stabilizing antiinflammatory activity of this fraction might be related to these biosynthesized metabolites.

## Conclusion

The crude methanol extract of the leaves of *C. citrinus* and its different organic fractions displayed different levels of free radical scavenging, thrombolytic and membrane stabilization activities. In the present study, the bioactive fractions were identified for particular cases. The crude methanol extract showed prominent free radical scavenging activity. On the other hand, the petroleum ether fraction displayed dominant thrombolytic action. However, the chloroform fraction was very effective for membrane stabilizing action. Further investigations are required to isolate bioactive compounds and know their detailed underlying mechanisms.

## Abbreviations

Not applicable.
